# Assessing safety climate in acute hospital settings: a systematic review of the adequacy of the psychometric properties of survey measurement tools

**DOI:** 10.1186/s12913-018-3167-x

**Published:** 2018-05-10

**Authors:** Gheed Alsalem, Paul Bowie, Jillian Morrison

**Affiliations:** 1 Institute of Health and Wellbeing, General Practice and Primary Care, University of Glasgow, 1,Horselethill Road, Glasgow, G12 9LX UK; 2 NHS Education for Scotland, 2 Central Quay, 89 Hydepark Street, Glasgow, Scotland G3 8BW UK; 3 Aramex House Old Bath Road Colnbrook, KWI 2656, Slough, Berkshire, SL3 0NS UK

**Keywords:** Attitudes of health personnel, Health care surveys/methods, Psychometrics/instrumentation, Surveys and questionnaires, Patient safety, Safety culture, Safety climate

## Abstract

**Background:**

The perceived importance of safety culture in improving patient safety and its impact on patient outcomes has led to a growing interest in the assessment of safety climate in healthcare organizations; however, the rigour with which safety climate tools were developed and psychometrically tested was shown to be variable. This paper aims to identify and review questionnaire studies designed to measure safety climate in acute hospital settings, in order to assess the adequacy of reported psychometric properties of identified tools.

**Methods:**

A systematic review of published empirical literature was undertaken to examine sample characteristics and instrument details including safety climate dimensions, origin and theoretical basis, and extent of psychometric evaluation (content validity, criterion validity, construct validity and internal reliability).

**Results:**

Five questionnaire tools, designed for general evaluation of safety climate in acute hospital settings, were included. Detailed inspection revealed ambiguity around concepts of safety culture and climate, safety climate dimensions and the methodological rigour associated with the design of these measures. Standard reporting of the psychometric properties of developed questionnaires was variable, although evidence of an improving trend in the quality of the reported psychometric properties of studies was noted. Evidence of the theoretical underpinnings of climate tools was limited, while a lack of clarity in the relationship between safety culture and patient outcome measures still exists.

**Conclusions:**

Evidence of the adequacy of the psychometric development of safety climate questionnaire tools is still limited. Research is necessary to resolve the controversies in the definitions and dimensions of safety culture and climate in healthcare and identify related inconsistencies. More importance should be given to the appropriate validation of safety climate questionnaires before extending their usage in healthcare contexts different from those in which they were originally developed. Mixed methods research to understand why psychometric assessment and measurement reporting practices can be inadequate and lacking in a theoretical basis is also necessary.

**Electronic supplementary material:**

The online version of this article (10.1186/s12913-018-3167-x) contains supplementary material, which is available to authorized users.

## Background

Patient safety in healthcare organizations has received much attention following the publication of the Institute of Medicine’s (IOM) report *“To Err Is Human: Building a Safer Health System”* in 2000. In its report, IOM highlighted the magnitude of preventable adverse events and identified the underlying “safety culture” as a key element influencing the ability of healthcare organizations to learn effectively from these events and implement preventative measures to reduce related harm to patients [1]. Assessing the status of the existing safety culture in a healthcare organization has been identified as the first step for developing a strong and solid safety culture [2]. Safety culture has been defined as “*the product of individual and group values, attitudes, perceptions, competencies, and patterns of behavior that determine the commitment to, and the style and proficiency of, an organization’s Health and Safety management*” ([3], p.23). According to Zohar [4], safety culture can be described as one aspect of an organization’s overall culture reflecting individual performance and organizational features that influence health and safety. Nevertheless, the concept remains poorly defined [5]. Pidgeon [6] has criticized earlier research for being “unsystematic, fragmented and in particular underspecified in theoretical terms” (p.203). Safety climate is often used interchangeably with safety culture [7] and can be perceived as “*the measureable components of safety culture*” ([8], p.364). It provides a “*snapshot*” of the perceptions and attitudes of the organization’s workforce about the surface-level aspects of culture during a particular point in time ([9], p.5). Safety culture and safety climate are clearly derivatives of organisational culture and climate [5, 10]. Researchers suggested that the concept of safety culture could be studied within the wider context of organisational culture [5, 11]. According to Neal and Griffin et al. [12], “*Safety climate is a specific form of organisational climate, which describes individual perceptions of the value of safety in the work environment*” (p.100). Reichers and Schneider [13] tracked the evolution of the two concepts and concluded that “*culture exists at a higher level of abstraction than climate, and climate is a manifestation of culture*” (p.23). In other words, safety culture is a broader organisational feature while safety climate is a sub-set of safety culture. Guldenmund [5] concludes that safety climate might be considered an alternative safety performance indicator. Cox and Flin [14] describe safety culture as the personality of an organization with its relative stability of systems, procedures and behaviours. Safety climate, on the other hand, was described as a transient mood state as changes in response to external events and pressures.

Ginsburg and Tregunno et al. [15] argue for the lack of clarity in defining the construct of safety culture and climate in addition to the construct of patient safety culture. It is, therefore, logical to suggest that the creation of a universal model or definition of safety culture is not straightforward [10]. Yet, it appears that most of the safety culture definitions across different organisations share essential elements including attitude and behaviour of workers in terms of health and safety performance [10]. These common elements indicate the psychological aspect of safety culture. This aspect refers to the highly related concept of safety climate. Safety climate is most commonly assessed by safety climate questionnaires to measure employee attitudes and perceptions of safety, as they are practical to apply in terms of time and cost-effectiveness [5, 16]. Cheyne and Oliver et al. [17] argue that these quantitative measurements apply only to a specific setting at a particular point in time and are subject to short-term fluctuations. Kirk and Parker et al. [18] add that despite their usefulness as safety measures, they offer a superficial evaluation of an organization’s culture. Additionally, Pronovost and Berenholtz et al. [19] demands the need for scientifically sound and feasible measures of patient safety. A range of questionnaire tools have been developed to assess safety climate in acute hospital settings, however the rigour with which they have been developed and psychometrically tested is variable [8, 20, 21]. A lack of rigorous psychometric evaluation makes it difficult to confirm the validity and reliability of survey scores and inform organisational learning and improvement. Thus, it is imperative that questionnaire tools are developed with robust psychometric properties [20].

Against this background, our systematic review aimed to identify and critically review the adequacy of the reported psychometric properties of tools designed to measure safety climate in acute hospital settings.

## Methods

### Search strategy

Electronic search of Medline, PubMed, CINAHL, Web of Science, PsycINFO, Embase and Scopus was performed, using the key terms: “Safety Culture”, “Safety Climate”, “Safety Attitudes”, “Hospital Safety”, “Patient Safety”, paired with “Health Care Workers”, with manual searches of bibliographies of included papers and key journals. This review covered English language studies published between January 2004 to December 2014. A detailed overview of the search strategies can be found in Additional file 1.

### Study selection

Two independent reviewers screened titles and abstracts of relevant records (GA, JM) while a third reviewer validated the data (PB).

#### Selection criteria

The process of identification and selection of eligible papers was conducted in two stages. The first stage involved evaluating the potential relevance of all titles and abstracts identified from the electronic database searches. Studies were included if they met the following criteria: (1) described a quantitative method of assessing patient safety climate, (2) described the results of tool development and psychometric evaluation, (3) directed at healthcare staff in a hospital setting. In the second stage, papers that were judged to be potentially relevant were retrieved and reviewed against the full text inclusion and exclusion criteria. To be eligible for inclusion at this stage, studies had to primarily focus on questionnaire development and explicitly state that the purpose of the study was to establish the psychometric properties of the tool as part of tool development, testing and implementation. Also, the tool also had to be designed for general administration to all healthcare staff working in a hospital setting and publically available. Only original tools developed in English-language were included as well as any updated version of an original tool that was produced by the original team in which the latest improved version was included.

**Fig. 1:**
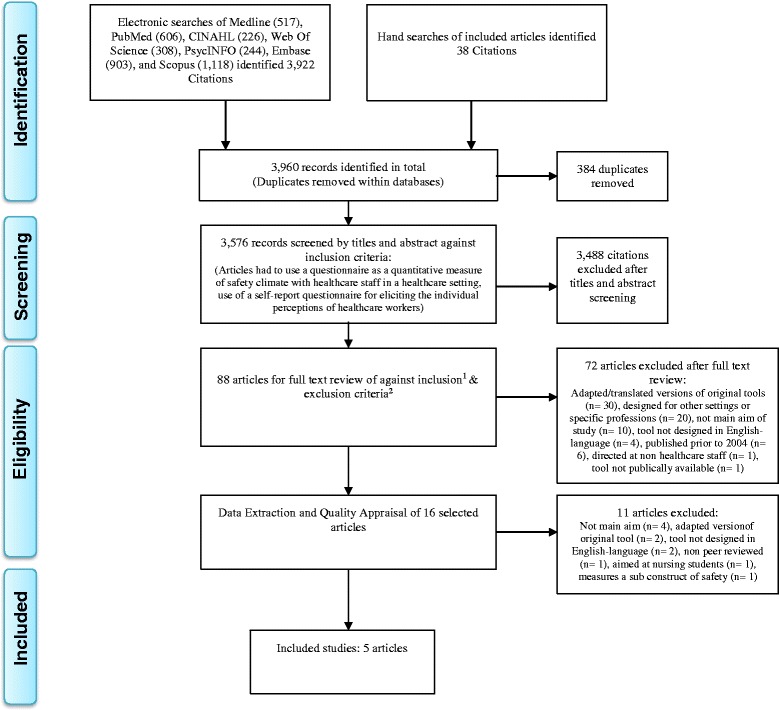
PRISMA Flow Diagram. Full Text Inclusion criteria^1^: Explicitly state the aim of the study is survey development & establishing the psychometric properties of the tool. Designed for general administration to healthcare staff in a hospital setting. Original tool or an updated version of an original tool produced by the original team. Questionnaires developed in English-language from January 2004 to December 2014. Publicly available tool. Full Text Exclusion criteria^2^: Qualitative studies. Opinion papers or grey literature. Target other aspects of culture. Adaptations of original survey tools. Designed for internal use in a single institution or for a particular specialty or profession. Aimed at junior doctors, nursing or medical students. Literature published prior to 2004

The flow chart in Fig. 1 shows the selection process, including the detailed inclusion and exclusion criteria.

#### Data extraction

Data extracted included a description of study setting; sample characteristics; study method; and tool features including dimensions covered, psychometric performed, theoretical basis and outcome measures (Tables 1, 2, 3).

**Table 1:** Data extraction results of general features

Features	Name of instrument				
	HSOPSC (1)	SAQ (2)	PSCHO (3)	SOS (4)	Can-PSC (5)
Authors	Sorra & Dyer	Sexton	Singer et al	Vogus & Sutcliffe	Ginsburg et al
Publication year	2010	2006	2007	2007	2013
Country	USA	USA	USA	USA	Canada
Instrument details: • Number of items • Type of Likert scale • Level of analysis • Results Reporting	42	60 (30 core items)	38	9	19
	5 point	5 point	5 point	7 point	5 point
	Individual, Unit, Hospital	Individual, Unit	Individual, Unit, Hospital	Unit	Unit, Hospital
	Positive percentage scores	Positive percentage scores	Percentage problematic scoring	Not reported	Not reported
Setting & Staff	Hospital setting Healthcare Staff	Hospital setting Healthcare Staff	Hospital setting Healthcare Staff including non clinical staff	Hospital setting Nursing units	Hospital setting Healthcare Staff

*HSOPSC* Hospital Survey on Patient Safety Culture, *SAQ* Safety Attitudes Questionnaire, *PSCHO* Patient Safety Climate in Healthcare Organizations, *Can-PSC* Canadian Patient Safety Climate Scale, *SOS* Safety Organizing Scale

**Table 2:** Data extraction results of specialized features

Features	Name of instrument				
	HSOPSC (1)	SAQ (2)	PSCHO (3)	SOS (4)	Can-PSC (5)
Safety Climate Dimensions: • Number of Dimensions Safety Climate Dimensions: • Scope of Dimensions	12	6	9	1	6
	Communication openness, Feedback and communication about error, Frequency of event reporting, Handoffs and transitions, Management support for patient safety, Non-punitive response to error, Organisational learning –Continuous improvement, Overall perceptions of patient safety, Staffing, Supervisor/manager expectations & actions promoting safety, Teamwork across units, Teamwork within units.	Teamwork, Safety climate, Job satisfaction, Stress recognition, Perception of management, Working conditions.	Senior manager’s engagement, Organisational resources for safety, Overall emphasis on safety, Unit safety norms, Unit recognition and support for safety efforts, Fear of shame, Provision of safe care, Learning, Fear of blame	Self-reported “behaviours enabling safety culture” through collective mindfulness.	Organisational leadership support for safety, Incident follow-up, Supervisory leadership for safety, Unit learning culture, Enabling open communication I: judgment-free environment, Enabling open communication II: job repercussions of error.
Theoretical basis	Literature review in areas of safety management; organizational & safety climate & culture; medical error & error reporting; patient safety. Existing safety climate and culture instruments.	Based on Vincent’s framework for analyzing risk & safety, Donabedian’s conceptual model for assessing quality Derived from an aviation safety culture questionnaire	High reliability organizations Derived from a naval aviation safety culture questionnaire	High Reliability organizations	Based on Zohar & Hofmann &Mark’s work on safety climate & error literature Adapted from work by Singer and colleagues
Key features	Tested on a large sample of hospitals Ability to benchmark data Self-report outcome measures	Tested on a large sample of hospitals Cross-industry comparisons Ability to benchmark data Favourable scores were associated with shorter lengths of stay& fewer medication errors in other studies	Measures safety climate among all hospital personnel and across multiple hospitals of different types Cross-industry comparisons	SOS is negatively associated with reported medication errors and patient falls	Validated for use across a range of care settings
Limitations	Supervisor/ Manager Expectations & Actions Promoting Patient Safety CFI =0.88 at unit & hospital levels Item A7 in the Staffing composite had a low within- unit & within hospital factor loading (0.36). Staffing had Cronbach’s alpha =0.62	(SRMR) model fit statistic at the clinical area level was larger than desirable, indicating further scale refinement Modest Response Rate	Three individual dimensions demonstrate low internal consistency. Selection Bias	Validated using a sample composed exclusively of registered nurses	Questions about generalizability Further research and cross- validation of will be required with international samples More appropriate for improvement and research Data was not suitable for multilevel CFA

*HSOPSC* Hospital Survey on Patient Safety Culture, *SAQ* Safety Attitudes Questionnaire, *PSCHO* Patient Safety Climate in Healthcare Organizations, *Can-PSC* Canadian Patient Safety Climate Scale, *SOS* Safety Organizing Scale

**Table 3:** Psychometric results

Features	Name of instrument				
	HSOPSC (1)	SAQ (2)	PSCHO (3)	SOS (4)	Can-PSC (5)
• Psychometric properties: • Content Validity • Construct Validity: ◦ Factor Structure • CFA ◦ Model fit indices^a^ • EFA ◦ Discriminant Validity • Criterion Validity • Reliability • Item analysis • Test Re-test Reliability • ANOVA	Yes	Yes	Yes	Yes	Yes
	Convergent Validity: CFA: 12 factors	Convergent Validity: CFA	Convergent Validity: MTA	Convergent Validity: CFA: Single factor	Convergent Validity: CFA
	CFI 5 out of 6 factors > 0.90 except^b^ SMEA	CFI 0.90 RMSEA 0.03	___	CFI > 0.90 RMSEA 0.06	CFI > 0.90 RMSEA 0.033
	SRMR < 0.08	SRMR 0.17 between & 0.04 within clinical areas		SRMR 0.033	–
	Chi < 0.05 Good model fit	Chi < 0.0001 Satisfactory model fit		Chi < 0.0001 Good model fit	Chi < 0.0001 Good model fit
	EFA: Yes 14 factors	EFA: Yes 6 factors	EFA: Yes 7 factors	EFA: No	EFA: Yes 6 factors
	Yes	Yes	Yes	Yes	Yes
	No	No	No	Yes	No
	Cronbach’s Alpha ≥0.70 except staffing	Raykov ñ coefficient = 0.90	Cronbach’s Alpha (0.50–0.89)	Cronbach’s Alpha ≥0.88	Cronbach’s Alpha (0.70–0.80)
	Yes	Yes	Yes	Yes	No
	No	No	No	No	No
	No	No	No	Yes	No

^a^Model fit indices recommended criteria: comparative fit index (CFI ≥0.90) [6], the standardized root mean square residual (SRMR < 0.08) & the Root Mean Square Error Of Approximation (RMSEA < 0.08) [7], Chi-square (Chi < 0.05) [8]

^b^Model fit indices were examined for six of the 12 HSOPSC composites that had four items. SMEA: Supervisor/manager expectations and actions promoting safety

#### Quality appraisal

Methodological quality of included studies was assessed based on the quality criteria developed by Flin and Burns et al. [20] (Table 4). Assessment of the quality of each study, included seven items related to the appropriateness of the study methodology, study population, data collection and analysis, response rate and results.

**Table 4:** Quality Appraisal Results

Quality Appraisal Criteria	HSOPSC Sorra and Dyer (2010) [23]	SAQ Sexton et al. (2006) [22]	PSCHO Singer et al. (2007) [24]	SOS Vogus & Sutcliffe (2007) [25]	Can-PSC Ginsburg et al. (2013) [15]
Aim(s) or research question(s) clearly stated?	✔	✔	✔	✔	✔
Study methodology and design evident and appropriate?	✔	✔	✔	✔	✔
Data collection described and appropriate?	✔	✔	✔	✔	✖
Study population described and appropriate?	✔	✖	✖	✔	✖
Data analysis method(s) described and appropriate?	✔	✔	✔	✔	✔
Response Rate acceptable (60% or above)	✖	✔	✖	✖	✔
Results reported in sufficient detail?	✔	✔	✔	✔	✔
Total Score	14/12	14/12	14/10	14/12	14/10
0–5 Poor Quality 6–10 Fair Quality 11–14 Good Quality Yes ✔, No ✖	Good	Good	Fair	Good	Fair

*HSOPSC* Hospital Survey on Patient Safety Culture, *SAQ* Safety Attitudes Questionnaire, *PSCHO* Patient Safety Climate in Healthcare Organizations, *Can-PSC* Canadian Patient Safety Climate Scale, *SOS* Safety Organizing Scale

Higher quality studies were considered to be those that met a minimum of six of these seven indicators.

Psychometric evaluation of included tools was based on recommendations by [20] and Flin and Burns et al. [20] and included aspects related to content, criterion and construct validity and reliability (Table 3).

Variability in safety climate dimensions across the reviewed papers have led the authors to evaluate the content of included dimensions in each tool. A list was developed including the most common safety climate dimensions that had been previously mentioned in studies addressing safety climate measures in healthcare (Table 5) [20]. Items and their suitability in each dimension were independently evaluated, by the three authors (GA, JM, PB), against the proposed list.

**Table 5:** Safety Climate Dimensions Categorization

Safety Climate Dimension	SOS items	HSOPSC Items	SAQ items	PSCHO items	Can-PSC items	Total Number of items/dimension	Percentage of items/dimension %
Top management support & institutional commitment to safety	0	7	6	9	7	29	20.6
Teamwork	5	8	7	0	0	20	14.2
Safety systems: “Policies&Procedures, Safety Planning, Hand offs & Transitions, Staffing, Equipment”	2	7	3	6	0	18	12.8
Safety perceptions & Attitudes of staff, Risk perceptions	0	3	3	9	1	17	11.3
Reporting Incidents & “non-punitive” response to error	0	3	1	6	5	14	10.6
Communication openness	1	4	4	1	0	10	7.1
Organizational learning and continuous improvement	1	3	0	1	4	9	6.4
Beliefs about the causes of errors & adverse events	0	0	4	3	0	7	5.0
Training & continuous education	0	0	3	2	0	5	3.5
Staff satisfaction	0	0	5	0	0	5	3.5
Feedback & Communication about adverse events	0	2	0	0	2	4	2.8
Work Pressure	0	2	0	1	0	3	2.1
Other	0	0	0	0	0	0	0.0
Total	9	39	36	38	19	141	100%

*HSOPSC* Hospital Survey on Patient Safety Culture, *SAQ* Safety Attitudes Questionnaire, *PSCHO* Patient Safety Climate in Healthcare Organizations, *Can-PSC* Canadian Patient Safety Climate Scale, *SOS* Safety Organizing Scale

This study updates an earlier review by Flin and Burns et al. [20] of quantitative studies of safety climate in healthcare aimed at examining their reported psychometric properties.

## Results

The search strategy identified a total of 3576 potential papers. Of these, 88 papers were reviewed against the full text inclusion criteria. Five studies met the criteria and were included for this review [15, 22–25] (Fig. 1). The tools included the Hospital Survey on Patient Safety Culture (HSOPSC) [23], Safety Attitudes Questionnaire (SAQ) [22], Patient Safety Climate in Healthcare Organizations (PSCHO) [24], Canadian Patient Safety Climate Scale (Can-PSC) [15], and the Safety Organizing Scale (SOS) [25]. The key features and characteristics of each included study and their reported psychometric properties are summarized in Tables 1, 2 and 3. Further information regarding each tool is in Additional file 1.

### General characteristics of reviewed studies

The five tools were designed for general assessment of patient safety climate in acute hospital settings. They aimed to assess respondents’ attitudes, perceptions and behaviors about various aspects of patient safety. They also sampled a variety of hospital personnel across different occupations, staff positions and work areas.

Four of the included tools originated from US studies [22–25] while one tool originated from a study in Canada [15].

All survey tools used Likert response scales. Length of survey tools ranged from nine to 60 questionnaire items with a total of 141 items distributed under 36 climate dimensions. Each tool covered between one (e.g. SOS) and twelve reported dimensions (e.g. HSOPSC).

Seven dimensions were addressed by the majority of the reviewed tools including: (1) Top management support, (2) Safety systems, (3) Safety attitudes of staff, (4) Reporting incidents, (5) Communication openness, (6) Organizational learning and (7) Teamwork (Table 5).

A number of tools were adapted from other industries. The SAQ, for example, is a an adaptation of a widely used questionnaire in the aviation industry [22]. More recently, tools have been developed specifically for healthcare settings such as the HSOPSC [23].

Four studies used theory to guide their tool development process. Within these studies, the PSCHO & SOS were based on High Reliability Organization Theory (HROT) [24, 25]. The SAQ employed more than one theory. Sexton and Helmreich et al. [22] Stated that the SAQ was based upon two conceptual models: Vincent’s framework for analyzing risk and safety [26] and Donabedian’s conceptual model for assessing quality [27]. Vincent’s framework incorporates the many factors influencing clinical practice including organizational factors and work environment factors while Donabedian’s conceptual model provides a framework for evaluating the quality of healthcare [26, 27]. The theoretical basis for the Can-PSCS is rooted in Zohar’s definition of safety climate and Hofmann and Mark’s model on safety climate [15]. Zohar’s definition of safety climate stresses management commitment to, and support of safety by leadership at multiple levels [28]. Hofmann and Mark’s model of safety climate emphasizes open communicating and constructive response to errors and the degree to which the social environment encourages these behaviours’ [29]. One study did not provide an explanation of the underlying theoretical basis [23].

### Methodological quality and psychometric assessment of reviewed studies

Convincing evidence of reliability and validity of any measuring tool can only be established by assessing the methodological quality of the studies. Our analysis focused on performing a comprehensive assessment of the reported psychometric properties in each study.

#### Methodological quality of reviewed studies

Three out of five studies [22, 23, 25] were rated as ‘good’ quality papers while two were rated as ‘fair’ [15, 24]. Studies that were rated as ‘good’, fulfilled six indicators related to: study aim(s), study methodology and design, data collection, study population, response rate, data analysis method(s) and results. The response rate fell below 60% for two of those studies [23, 25]. One study did not report their study population in sufficient detail to allow judgment to be made [22].

Papers rated as ‘fair’ quality, including Singer and Meterko et al. [24] and Ginsburg and Tregunno et al. [15], did not describe their study population in sufficient detail. The response rate was not acceptable in PSCHO while data collection was not sufficiently described in Can-PSC. The quality appraisal results for each individual study are summarized in Table 4.

#### Psychometric properties of reviewed instruments

The psychometric properties of included safety tools were examined with respect to content validity, criterion validity, construct validity (EFA, CFA) in addition to reliability (Table 6). Other measure included correlation across dimensions, item analysis, test/retest reliability and analysis of variance. All of the reviewed tools covered the standard psychometric criteria, as recommended by Flin and Burns et al. [20] (Table 3). However, three tools, including the HSOPSC, SAQ and SOS, reported more robust psychometric properties following their psychometric assessment in comparison to PSCHO and Can-PSCS.

**Table 6:** Psychometric Properties

Content Validity	
Haynes et al. (1995, [77] p.238) defined Content Validity as “the degree to which elements of an assessment instrument are relevant to and representative of the targeted construct for a particular assessment purpose”. It is used for ascertaining whether the content of the measure was appropriate and pertinent to the study purpose and is usually undertaken by seven or more experts in addition to other sources including review of empirical literature and relevant theory [78].	
Criterion Validity	
Criterion validity delivers evidence about how well scores on a measure correlate with other measures of the same construct or very similar underlying constructs that theoretically should be related [79]. As Flin et al. (2006) [20] indicated, Criterion Validity could be established by correlating the safety climate scores with outcome measures. Outcome measures of safety in health care could include items such as patient injuries, worker injuries, or other organizational outcomes [20].	
Construct Validity	
Construct validity can be defined as the degree to which items on an instrument relate to the relevant theoretical construct [80]. A variety of ways exists to assess the construct validity of an instrument, including Factor analysis. Factor analysis is a statistical method that “explores the extent to which individual items in a questionnaire can be grouped together according to the correlations between the responses to them”, thus reducing the dimensionality of the data (Hutchinson et al., 2006, [81] p.348). Convergent Validity represents the degree to which different measures of the same construct show correlation with each other and is tested using confirmatory factor analysis (CFA). Conversely, Discriminant Validity represents the extent to which measures of different constructs show correlation with one other [78]. The two main techniques of Factor Analysis are Exploratory Factor Analysis (EFA), and Confirmatory Factor Analysis (CFA). EFA is used to uncover the underlying factor structure of a questionnaire, while CFA is used to test the proposed factor structure of the questionnaire [81]. A CFA measurement model shows convergent validity if items load significantly (.40 or greater) onto the assigned factor and model fit indices suggest adequate fit [25]. Models with a cutoff value close to .90 for CFI; a cutoff value close to .08 for SRMR; and a cutoff value close to .06 for RMSEA are indicative of good model fit [38].	
Reliability	
Reliability reflects the degree to which test scores are replicable [76, 82]. It ensures that respondents are responding consistently to the items within each composite. Reliability is also referred to as consistency. It can be assessed using Cronbach’s alpha, which is the most commonly used internal consistency reliability coefficient. Cronbach’s alpha ranges from 0 to 1.00 with the minimum criterion for acceptable reliability is an alpha of at least .70. [83, 84].	

The quality appraisal results of each survey tools’ psychometric properties are shown in Table 3.

### Content validity

Instrument development, in all studies, typically involved the use of literature reviews, opinions of safety experts and user populations to conceptualize the domains of safety culture to be measured, and to generate related questionnaire items. Definitions of safety climate and culture overlapped among the studies although two studies clearly draw a distinction between the two terms and stressed that they set out to measure safety climate [15, 22].

Regarding the theoretical basis of the tools, three studies [15, 22, 25] stated that their survey items were based on a conceptual model but it was not clear how they related theory to their questionnaire items. One exception was the PSCHO where an explanation of its nine-dimension theoretical model was provided [24]. The HSOPSC had no explicit theoretical basis [23].

Two “core” safety dimensions from the non-healthcare industrial sector, ‘management and supervisory commitment to safety’ and ‘safety systems’, were measured in four studies as components of safety climate in healthcare [20] (Table 2). A plausible explanation is that most of the instruments were based on High Reliability Organization Theory or were derived from tools designed for those specific industries such as the SAQ.

### Criterion validity

Three studies had no reported independent outcome measures of safety climate [15, 22, 24]. The HSOPSC included two self-reported outcome measures: ‘Patient Safety Grade’ and ‘Number of Events Reported’. Positive associations have been shown between climate scores and self-reported safety measures [23].

A single study used independent measures to examine significant associations between safety climate scores and outcomes where multilevel regression analysis showed a negative relationship between SOS and reported medication errors and patient falls [25].

### Construct validity

#### Factor structure and internal reliability

All five studies reported the results of a factor analysis (Table 3). CFA was performed in four studies [15, 22, 23, 25]. CFA results are evaluated by examining the items factor loadings (0.40 or greater) and the overall model fit indices.

The HSOPSC reported factor loadings ranging from 0.36 to 1.00. The staffing composite had one item with low factor loadings (0.36). The model fit indices were good with the exception of *Supervisor/Manager Expectations and Actions Promoting Patient Safety* which reported comparative fit indicator (CFIs) below the recommended 0.90 criterion.

SAQ reported factor loadings ranging from 0.40 to 0.99. The overall model fit indices were good. The SOS and the Can-PSC scales reported good model fit indices as well. PSCHO did not report any model fit indices.

EFA was reported to be performed in all studies except the SOS [25].

Reliability was reported in all of the studies (Table 3) and internal consistency (Cronbach’s Alpha) was reported in four out of five studies and exceeded the accepted standard (≥ 0.70), in the majority of the scale composites. The only two exceptions were the HSOPSC (Staffing α = 0.62) and the PSCHO (Learning α =0.50, Fear of shame α =0.58, Fear of blame α =0.61). Raykov’s ñ coefficient was reported as the scale reliability estimate for the SAQ [22]. Raykov’s ñ coefficient value was 0.90, indicating strong scale reliability.

#### Intercorrelations

The HSOPSC intercorrelations, both among and between the 12 safety composites and the tool’s two outcome measures, were moderate [23]. SAQ’s reported intercorrelations were significant with a few exceptions [22]. PSCHO reported results confirm that the measure reflects correlated but distinct aspects of safety climate [24]. The Can-PSC showed that discriminant validity was supported for all dimensions with the exception of the incident follow-up dimension [15].

## Discussion

This study aims to provide a comprehensive review of quantitative studies designed to assess safety climate in the hospital setting, with particular focus on questionnaires. The objective of the systematic review was to provide a structured overview of their psychometric adequacy as measurement tools for their stated purpose.

All of the five reviewed safety climate tools have key similarities and common dimensions. Yet, they vary in terms of length, theoretical grounding and reported psychometric properties. Instruments varied in scope, with some covering a more comprehensive range of dimensions while others focused on the assessment of specific dimensions of safety culture. For example, the HSOPSC is a broad 42 items’ tool that covers twelve safety culture composites and is directed at a wide range of specialties and different care settings. It is arguably more suitable for a patient safety interventional programme as it may have greater potential for uncovering areas in need of improvement compared to shorter questionnaires like the one dimensional 9-item SOS. As a result, this tool may be less sensitive in identifying problematic areas [30]. Shorter questionnaires, however, have the potential to increase the response rate and reduce the non-response bias associated with longer surveys [23, 31].

### Psychometric properties

Despite the growing body of evidence about the value of establishing the psychometric properties of safety climate tools, there is still a lack of proper reporting of related questionnaire properties across published literature [8, 20, 32, 33]. Studies have shown considerable variation regarding the methods and the standards applied in reporting the psychometric properties [34]. This can be partly explained by the methodological rigor and resources required for safety climate tools to be appropriately developed and psychometrically tested [21].

Flin and Burns et al. [20] proposed that tools must be developed with robust psychometric properties to confirm the validity and reliability of safety climate test scores and enable proper identification of underlying dimensions.

Emerging evidence about the predictive validity of safety climate measures suggest that positive safety climate scores are associated with clinical outcomes including shorter lengths of stay and fewer medication errors [21]. Favourable scores have been linked also to safety-related behaviours and attitudes of healthcare staff [4, 35, 36]. Thus, in order to provide reliable data, it is imperative that tools are developed with robust psychometric properties that enable valid interpretations of patient safety climate test scores [20].

Colla and Bracken et al. [8] and Flin and Burns et al. [20] argued that there was a limitation on reporting the psychometric properties for most of their reviewed safety climate tools. Two notable exceptions were the HSOPSC and the SAQ where more of the indicated psychometric criteria were met. Conversely, Perneger and Staines et al. [37] argue that even the original HSOPSC instrument did not fulfill the standard psychometric criteria of a sound structure as proposed by Hu and Bentler [38] and recommended that the instrument be partially redesigned.

In comparison to earlier studies, where standard psychometric criteria were not reported [12, 39–42], our study showed that all of the reviewed tools covered the standard psychometric criteria, as recommended by Flin and Burns et al. [20]. This provide evidence for an improving trend in reporting the psychometric properties of tools in this area. This, as a result, places safety climate assessment on the right track.

A number of reported adaptations of the HSOPSC, in China, France, Norway and the UK [37, 43–46], have performed less well than the original tool. This might be due to the contextual specificity of the construct of safety culture [47]. As a result, there is a need for appropriate validation of safety climate questionnaires before extending their usage in healthcare contexts different from those in which they were originally developed [34].

### Safety climate dimensions

Over the past 10 years, a number of comprehensive reviews of studies addressing patient safety in general or patient safety climate instruments in particular have been conducted [8, 20, 21, 48–52]. Most studies have suffered from an absence of clarity in defining the constructs of safety culture and climate in addition to that of patient safety culture [15]. The construct of safety culture has been described by Reason [53] as having the “definitional precision of a cloud” (p.192). This is reflected in a wide range of dimensions being incorporated into safety climate surveys, which may “dilute this domain” ([54], p.2). A significant degree of overlap exists in the content of the dimensions between different surveys (e.g. between the items within dimensions related to teamwork and communication openness), which may be a consequence of using different definitions (broad or narrow) of safety dimensions. Differences in judgment of the content of dimensions between different authors also play a major role. As a results, it is difficult to judge whether measures exploring twelve dimensions have greater or lesser validity than those measures examining only one dimension [21].

The most common dimensions mentioned in the above review studies were used as the basis for our categorization process. Our results show an overlap with those reviews as seven of the included dimensions were covered by our five reviewed tools (Table 7). The results also corroborate the recommendations of Singla and Kitch et al. [21], which suggested that common dimensions including communication, teamwork, and leadership support might be considered “core dimensions” of patient safety culture.

**Table 7:** A comparison of the common safety climate dimensions in healthcare that are mentioned in four review papers

Safety culture dimensions	Safety climate/culture studies					
	Colla and Bracken et al. [8] 9 Tools	Flin and Burns et al. [20] 12 Tools	Singla and Kitch et al. [21] 13 Tools	Fleming and Wentzell [52] 4 studies	Halligan and Zecevic [49] 130 Studies	Current systematic review
Top management support	√	√	√	√	√	√
Teamwork		√	√	√	√	√
Safety systems	√	√		√		√
Feedback & Communication	√	√		√		
Reporting Incidents	√	√		√	√	√
Communication openness		√	√		√	√
Organizational learning				√	√	√
Beliefs about the causes of errors & adverse events			√			
Work Pressure		√		√		
Risk perception		√		√		
Beliefs about the importance of safety					√	
Safety Attitudes of staff		√				√

In our view, the SAQ addresses human factors and job satisfaction along with fundamental aspects of safety culture while the HSOPSC includes handoffs and transitions and the role of supervisors in promoting patient safety. Risk-taking behavior, a commonly measured safety dimension in other industries, was only covered by the PSCHO [24]. The SOS was developed as a self-report measure of safety organizing that captures the behaviours theorized to enable a safety culture [25]. It mainly stresses teamwork. Ausserhofer [55] highlighted that the SOS items, compared to the SAQ, might not fully capture the “psychological safety” aspects including fear of blame and shame (p.131). The Can-PSCS focuses on management commitment to patient safety and is recommended for use before patient safety improvement initiatives focusing on learning from errors in order to assess the context for change. Finally, the PSCHO focuses on management commitment to safety, safety systems, and safety attitudes of staff. (Table 2). This diversity in focus is partly related to the tools’ development process as the above models are mainly driven by expert opinion and not necessarily reflect what hospital staff think about patient safety [37].

Most of the reviewed studies failed to examine the influence of local cultural factors as part of their safety climate assessment tools. Almutairi [56] questioned the impact of multicultural workforce on safety climate in healthcare settings and concluded that this diversity can adversely affect the quality of care and patient safety. In a study by Algahtani [57], the author investigated the influence of a multicultural workforce in Saudi Arabia on patient safety and developed a new dimension, *Multicultural Workplace*, with items relating to local culture to assist in measuring cultural factors related to patient safety. Results showed strong, positive correlations with most SAQ dimensions indicating its relevance and importance to the safety culture. Another area that is overlooked is the physical environment of a hospital facility, including its technology and equipment, and its effect on patient safety [58]. In addition, little is known about the effect of the psychosocial work environment, including job demands and resources available to cope with them, on safety climate [59].

Overall, these studies highlight the need for robust research to clarify which dimensions belong to the core concept of safety culture, as Flin and Burns et al. [20] have argued for, “a set of universal or core variables that underpin safety climate across work sectors”.

### Theoretical basis

The lack of theoretical evidence supporting the process of safety climate tools’ development has been clearly articulated by most of the reviews carried out in industry and in healthcare [8, 20, 60]. A theoretical basis is deemed to be an essential component of a psychometrically sound tool to outline the proposed relationships between safety climate and safety outcomes and if theoretical assumptions are not explicit, then evidence on the construct validity of the developed instruments is inadequate. This makes it difficult for organizations to use questionnaires effectively for organizational learning and development aims [49, 61].

Guldenmund [5] conducted a review of safety culture and climate research and concluded that “All in all, the models of safety culture are unsatisfactory to the extent that they do not embody a causal chain but rather specify some broad categories of interest and tentative relationships between those” (p243). Additionally, Groves and Meisenbach et al. [62] argue that it is not surprising for a concept, such as safety culture, collected from multiple disciplines, to lack a strong theoretical basis in a fundamentally different healthcare setting.

The theoretical roots of patient safety culture research lie mostly in high-risk industries [55]. According to Halligan and Zecevic [49], the five most commonly cited theories or models in healthcare research include: [1] High-Reliability Organization Theory (HROT) [2, 63] Donabedian’s Quality of Care Model [64] and its adaptations including the SEIPS model [58] and Quality Health Outcomes Model [3, 65] The Cultural Maturity Model [4, 66] Organizational Theory [63] and [5] System Theory [67]. According to Guldenmund [5]), no single safety culture theory or model has been universally accepted as clearly reflecting the construct of safety culture and safety climate and none of the theories or models may be applicable to all types of organisations .

Safety culture and climate theories in healthcare are clearly limited as none of the reviewed tools draw upon related theory. Additionally, it was stated earlier that four studies used theory to guide their tool development process but none provide an adequate explanation about their guiding theoretical framework nor do they clearly articulate the links between questionnaire items and specific theoretical constructs. Walshe and Boaden [68] point to the HSOPSC as having “no explicit theoretical framework”. HSOPSC, along with SAQ, was developed based on literature reviews, existing safety culture instruments and further input by researchers and hospital administrators. This suggests that researchers overlooked the importance of the epistemological and theoretical roots that underlie the development of their instruments [49] with more focus on the measurement rather than further conceptual development [69].

Groves and Meisenbach et al. [62] add that recent developments in safety culture have heightened the need for a theory that describes the process of keeping patients safe through the interaction between organizational structures at the macro-level and individual actions at the micro-level. They add that such theory is crucial for further progress towards patient safety.

Reiman and Silla et al. [61] stated that most studies overlook defining the underlying concept and frequently jump directly to “operationalisation” of the measure. This makes it difficult to evaluate how well the questionnaire actually measure the phenomena it aims to measure.

Early attempts to measure safety climate were based on adapting existing instruments from other industries (aviation, oil, nuclear) to healthcare settings [70, 71]. For example, the PSCHO and SOS were based on HROT. The theoretical bases of some of the original instruments, explain relationships between safety culture or safety climate in settings “*far-removed from healthcare*” ([55], p.129). The organisational structures and cultures of such industries are different than those of healthcare organisations [72]. When the processes of safety culture are not clearly understood, this makes it difficult to evaluate how well the questionnaire actually measures the phenomena under study and calls into question the interpretation of survey results [62].

In 2006, Flin et al. reviewed twelve safety climate measures designed for the health care setting. Building on their search criteria, we have uncovered additional measures being applied for different healthcare settings. Nevertheless, these additional measures, arguably, add to the present state of ambiguity in the assessment of safety culture in healthcare.

In this review, a detailed inspection of the included tools revealed a number of limitations to these measures. The limitations and ambiguity center around the concepts of safety culture and climate, their associated dimensions, the methodological rigor associated with the design of these measures and the lack of clarity in the relationship between safety culture and outcomes [62]. The influence of safety climate on patient and worker safety outcomes is not yet clear, though studies have started to confirm that safety climate scores can be associated with healthcare workers’ safety behaviours or workers’ injuries [32]. There is a need for more evidence to understand how the use of safety culture or climate tools impact on outcomes [73].

In a number of comprehensive reviews of safety climate tools in healthcare, the HSOPSC and SAQ repeatedly emerged as recommended tools [20, 21, 48, 49]. Results of our systematic review seem to mirror findings of previous studies that have examined hospital safety climate where the three studies that reported the SAQ, HSOPSC and SOS tools have been reported to have good assessment of their reported psychometric properties [22, 23, 25].

### Strengths and limitations

The strengths of our review are that it represented a comprehensive examination of safety climate tools designed for hospitals. A thorough search strategy was employed with all stages of the review process performed with at least two independent reviewers in order to avoid selection bias. Study rigor was enhanced using a pre-set protocol, standardized forms, and a series of indicators to assess the quality of the reviewed studies and the reported psychometric properties.

There are also several limitations to our study. The exclusion of other bibliographic databases, grey literature, and non- English language papers could potentially lead to overlooking some studies. Regarding the assessment of the quality of the reviewed studies, some quality indicators were not reported in sufficient detail to allow a judgment to be made. In such cases, the indicator was marked as unmet and the study quality might have been underestimated due to under reporting. Finally, despite using three reviewers to categorise the items, there is still the possibility that bias was introduced by the qualitative nature of the process.

### Theoretical and practical implications

Further research is necessary in the development of safety culture theories in healthcare, to study the links between culture and outcomes, and to resolve the controversies in the definitions and dimensions of safety culture and climate [74]. There is also a need for a safety climate tool to evaluate safety attributes in the “local” hospital setting bearing in mind the unique characteristics of that particular setting and population.

On a practical level, the development of a standardized checklist for assessing the quality of climate questionnaires, including reported psychometrics, may be beneficial and help provide a more detailed account of the questionnaire development process. Additionally, employing mixed methods tool development approaches may help to reveal different aspects of an organization’s safety culture, which can inform and illuminate multiple components of this multidimensional construct than is currently the case [50].

## Conclusions

The perceived importance of safety culture in improving patient safety and its impact on patient outcomes has led to an increasing number of studies that attempt to define and assess safety culture in healthcare settings. Several reviews uncovered a wide variety of safety climate tools available for use [8, 20, 21]. Still, theoretical and methodological challenges limit their use as assessment measures. Pronovost and Sexton [75] warned that “the enthusiasm for measuring culture may be outpacing the science”. Critics have increasingly called for more rigorous assessments of safety culture and more in-depth reporting.

It is recommended that research first be conducted to resolve the controversies in the definitions and dimensions of safety culture and climate, and focus on developing theoretical models with more evidence to understand how safety culture or climate impacts on outcomes. Also, more consideration should be given to psychometric properties in the design and selection of tools in order to ensure the robustness of the resulting safety culture data.

Psychometric testing, on its own, does not fully characterize an instrument with other forms of item analysis, such as cognitive testing, as they provide rich insight into locally held attitudes and perceptions related to patient safety.

When choosing a suitable instrument, healthcare providers should be guided by a combination of factors including intended purpose, target population, and the tool’s reported psychometric properties. This is likely to be an identified training need for those interested in understanding of the differences between the various available instruments and their limitations. The outcomes of this systematic review will provide guidance and support to healthcare policymakers, survey users and safety researchers to make more informed decisions when selecting or developing an appropriate safety climate assessment tool.

## Additional file

Additional file 1: Search Strategy. Electronic databases search strategy. Tools Descriptions. Descriptions of the five tools examined in the systematic review. (DOCX 42 kb)

## Abbreviations

Can-PSC: Canadian patient safety climate scale
CFA: Confirmatory factor analysis
CFI: Comparative fit index
Chi: Chi-square
EFA: Exploratory factor analysis
HROT: High reliability organization theory
HSOPSC: Hospital survey on patient safety culture
IOM: Institute of medicine’s
MCFA: Multilevel CFA
PSCHO: Patient safety climate in healthcare organizations
RMSEA: Root mean square error of approximation
SAQ: Safety attitudes questionnaire
SOS: Safety organizing scale
SRMR: Standardized root mean square residual
