# Supplementary material for: Assessing safety climate in acute hospital settings: a systematic review of the adequacy of the psychometric properties of survey measurement tools
Source: BMC Health Serv Res. 2018 May 10;18:353. doi: 10.1186/s12913-018-3167-x (PMC5946435; doi:10.1186/s12913-018-3167-x)
Supplement: Supplementary file 1 — Search Strategy. Electronic databases search strategy. Tools Descriptions. Descriptions of the five tools examined in the systematic review. (DOCX 42 kb) [file 12913_2018_3167_MOESM1_ESM.docx]

***Search Strategy***

Database: Ovid MEDLINER In-Process & Other Non-Indexed Citations and Ovid MEDLINER <1946 to Present>

Search Strategy:

--------------------------------------------------------------------------------

1 safety adj3 culture or climate or attitude or patient or hospital.mp. [mp=title, abstract, original title, name of substance word, subject heading word, keyword heading word, protocol supplementary concept word, rare disease supplementary concept word, unique identifier] 22995

2 Personnel, Hospital/ or Health Personnel/ 37131

3 1 and 2 576

4 limit 3 to english language and yr="2000 -Current" 504

| **Ovid MEDLINER In-Process & Other Non-Indexed Citations and Ovid MEDLINER** 1946 to Present |
| --- |

| 1 | safety adj3 culture or climate or attitude or patient or hospital.mp. [mp=title, abstract, original title, name of substance word, subject heading word, keyword heading word, protocol supplementary concept word, rare disease supplementary concept word, unique identifier] |
| --- | --- |
| 2 | Personnel, Hospital/ or Health Personnel/ |
| 3 | 1 and 2 |
| 4 | limit 3 to english language and yr="2000 -Current" |

**Database: Ovid MEDLINER In-Process & Other Non-Indexed Citations and Ovid MEDLINER <1946 to Present> Search Strategy:**

|  | safety.ti,ab. | 324225 |
| --- | --- | --- |
|  | safe.ti,ab. | 248872 |
|  | 1 or 2 | 518103 |
|  | exp organizational culture/ or exp culture/ | 135046 |
|  | culture.ti,ab. | 476907 |
|  | 4 or 5 | 597218 |
|  | patient$ adj2 safety.ti,ab. | 20852 |
|  | safety adj2 culture.ti,ab. | 1433 |
|  | safety adj2 climate.ti,ab. | 527 |
|  | safe$ adj2 attitude.ti,ab. | 104 |
|  | hospital adj2 safety.ti,ab. | 828 |
|  | 7 or 8 or 9 or 10 or 11 | 22447 |
|  | Health Personnel/ | 25900 |
|  | 3 and 6 and 13 | 136 |
|  | 12 and 13 | 366 |
|  | 14 or 15 | 427 |
|  | limit 16 to english language and yr="2000 -Current" | 387 |

**PubMed Search Strategy Summary:**

(Attitude of Health Personnel [MeSH Terms]) AND ((((((safety climate[Title/Abstract]) OR safety culture[Title/Abstract]) OR safety attitude[Title/Abstract]) OR patient safety[Title/Abstract]) OR hospital safety[Title/Abstract]))

**CINAHL and PsycINFO Search strategy Summary:**

AB Safety N3 Culture OR climate OR attitude OR patient OR hospital AND SU health personnel

**Scopus Search Strategy Summary:**

(TITLE-ABS-KEY (Culture OR climate OR attitude OR patient OR hospital)) AND (TITLE-ABS-KEY (health personnel))

**Web Of Science Search Strategy Summary:**

TOPIC: ((safety) NEAR/3 (culture or climate or attitude or patient or hospital)) AND TOPIC: (health personnel)

**Acknowledgements:**

- Not available

***Tools Descriptions***

***The Hospital Survey on Patient Safety Culture***

The HSOPSC can be viewed as a comprehensive measure of safety climate in healthcare settings. Since its introduction in 2004, the HSOPSC has been validated for use in more than 60 countries and translated to 30 different languages (1, 2, 3, 4, 5, 6, 7, 8). The HSOPSC includes 42 items that assess twelve dimensions of patient safety culture at the individual, unit and hospital levels. Also, four outcome measures are included in the tool. Results from the HSOPSC can be used to assess the current status of safety climate and study the impact of patient safety initiatives and programmes. It also assists in benchmarking against other hospitals and fulfil regulatory requirements.

***The Safety Attitudes Questionnaire***

The Safety Attitudes Questionnaire (SAQ) has been derived from a widely used questionnaire in aviation (9, 10, 11). The original version of the SAQ consists of 60 items including 30 core items that are the same in all clinical settings. It measures healthcare providers’ attitudes about six safety-related dimensions at both the individual and group level (12). Thus, clinical areas can benchmark their climate against themselves and against other units in their organizations (Sexton et al., 2006, p.44). Also, it helps in assessing the impact of initiatives implemented to improve patient safety (13).The SAQ has been used in different settings (12) and has been translated into different languages (10). The SAQ has been used to investigate the relationship between safety climate scores and patient outcomes as favorable scores were found to be associated with shorter lengths of stay and less medication errors (13).

***The Safety Organizing Scale***

The Safety Organizing Scale (SOS), developed by Vogus & Sutcliffe in 2007, is a promising tool that assesses the degree to which healthcare staff are involved in safety behaviors and practices in their units. The questionnaire items were developed based on the five inter-related behavioral processes of “collective mindfulness” (Vogus & Sutcliffe, 2007, p.47) . The development of the SOS was based on case studies of “high-reliability organizations” (HROs).

***Patient Safety Climate in Healthcare Organisations***

The PSCHO tool was developed as part of a Stanford-based patient safety research program (15). HROT guided development of the PSCHO. It was one of the first tools developed to measure safety climate among all hospital personnel and across multiple hospitals of different types. PSCHO items are spread over nine constructs, three organisational factors, two unit factors, three individual factors and one additional factor. The tool has been used for assessing safety climate in hospitals largely in the US.

***Can-PSCS***

The revised Canadian Patient Safety Climate Survey (Can-PSCS) provides a measure of direct care providers’ perceptions of patient safety climate (16). The Can-PSCS is designed and validated for use across a variety of care settings with robust psychometric properties. The theoretical model for the Can-PSCS is based on Zohar’s and Hofmann and Mark’s work on safety climate. The Can-PSCS contains 19 items designed to reflect six dimensions. Dimensions of organisational leadership for safety and supervisory leadership support for safety are based on Zohar’s definition of safety climate as management commitment to, and support of safety by leadership at multiple levels (17). Dimensions of patient safety learning culture and enabling communication about errors are supported by Hofmann and Mark’s model of safety climate, which emphasises open communicating and constructive response to errors and the degree to which the social environment encourages these behaviours’ (18). Although the Can-PSCS reported good psychometrics, Ginsburg and Tregunno et al. (16) recommend further improvements to the tool.

**References:**

1. Sarac C, Flin R, Mearns K, Jackson J, editors. Measuring hospital safety culture: Testing the HSOPSC scale. Proceedings of the Human Factors and Ergonomics Society; 2010.

2. Bodur S, Filiz E. Validity and reliability of Turkish version of "hospital Survey on Patient Safety Culture" and perception of patient safety in public hospitals in Turkey. BMC Health Services Research. 2010;10.

3. Pfeiffer Y, Manser T. Development of the German version of the Hospital Survey on Patient Safety Culture: Dimensionality and psychometric properties. Safety Science. 2010;48(10):1452-62.

4. Perneger TV, Staines A, Kundig F. Internal consistency, factor structure and construct validity of the French version of the Hospital Survey on Patient Safety Culture. BMJ quality & safety. 2014;23(5):389-97.

5. Haugen AS, Søfteland E, Eide GE, Nortvedt MW, Aase K, Harthug S. Patient safety in surgical environments: cross-countries comparison of psychometric properties and results of the Norwegian version of the Hospital Survey on Patient Safety. BMC health services research. 2010;10(1):1.

6. Smits M, Christiaans-Dingelhoff I, Wagner C, Wal G, Groenewegen PP. The psychometric properties of the 'Hospital Survey on Patient Safety Culture' in Dutch hospitals. BMC Health Services Research. 2008;8:230.

7. Waterson P, Griffiths P, Stride C, Murphy J, Hignett S. Psychometric properties of the Hospital Survey on Patient Safety Culture: findings from the UK. Quality and Safety in Health Care. 2010;19(5):e2-e.

8. Agency for Healthcare Research and Quality. International Use of the Surveys on Patient Safety Culture Rockville, MD2015 [Available from: <http://www.ahrq.gov/professionals/quality-patient-safety/patientsafetyculture/pscintusers.html>.

9. Helmreich R, Merritt A, Sherman P, Gregorich S, Wiener E. The flight management attitudes questionnaire (FMAQ). Austin, TX: University of Texas 1993. Contract No.: NASA/UT/FAA Technical Report 93-4.

10. Sexton J, Helmreich R, Neilands T, Rowan K, Vella K, Boyden J, et al. The safety attitudes Questionnaire: psychometric properties, benchmarking data, and emerging research. BMC Health Services Research. 2006;6:44.

11. Sexton JB, Thomas EJ, Helmreich RL. Error, stress, and teamwork in medicine and aviation: cross sectional surveys. Bmj. 2000;320(7237):745-9.

12. Pronovost P, Sexton B. Assessing safety culture: guidelines and recommendations. Quality and safety in health care. 2005;14(4):231-3.

13. Thomas EJ, Sexton JB, Neilands TB, Frankel A, Helmreich RL. The effect of executive walk rounds on nurse safety climate attitudes: a randomized trial of clinical units. BMC health services research. 2005;5(1):1.

14. Vogus TJ, Sutcliffe KM. The safety organizing scale: development and validation of a behavioral measure of safety culture in hospital nursing units. Medical care. 2007;45(1):46-54.

15. Singer S, Meterko M, Baker L, Gaba D, Falwell A, Rosen A. Workforce perceptions of hospital safety culture: development and validation of the patient safety climate in healthcare organizations survey. Health Services Research. 2007;42(5):1999-2021.

16. Ginsburg LR, Tregunno D, Norton PG, Mitchell JI, Howley H. ‘Not another safety culture survey’: using the Canadian patient safety climate survey (Can-PSCS) to measure provider perceptions of PSC across health settings. BMJ quality & safety. 2013:bmjqs-2013-002220.

17. Zohar D. Safety climate in industrial organizations: theoretical and applied implications. Journal of applied psychology. 1980;65(1):96.

18. Hofmann DA, Mark B. An investigation of the relationship between safety climate and medication errors as well as other nurse and patient outcomes. Personnel Psychology. 2006;59(4):847-69.
